# Targeting liver cancer stem cells: the prognostic significance of MRPL17 in immunotherapy response

**DOI:** 10.3389/fimmu.2024.1519324

**Published:** 2024-12-17

**Authors:** Jingjing Shao, Tianye Zhao, Jibin Liu, Peipei Kang

**Affiliations:** ^1^ Cancer Research Center Nantong, Affiliated Tumor Hospital of Nantong University & Nantong Tumor Hospital, Nantong, China; ^2^ Department of Anesthesiology, Affiliated Tumor Hospital of Nantong University & Nantong Tumor Hospital, Nantong, China; ^3^ Department of Anesthesiology, First Affiliated Hospital of Soochow University, Suzhou, China

**Keywords:** cancer stem cell, hepatocellular carcinoma, single cell analysis, MRPL17, machine learning

## Abstract

**Background:**

Liver hepatocellular carcinoma (LIHC) ranks as the foremost cause of cancer-related deaths worldwide, and its early detection poses considerable challenges. Current prognostic indicators, including alpha-fetoprotein, have notable limitations in their clinical utility, thereby underscoring the necessity for discovering new biomarkers to improve early diagnosis and enable personalized treatment options.

**Method:**

This investigation employed single-cell analysis techniques to identify stem cell-associated genes and assess their prognostic significance for LIHC patients, as well as the efficacy of immunotherapy, utilizing nonnegative matrix factorization (NMF) cluster analysis. A diagnostic model for LIHC was developed and validated through multiple datasets and various machine learning clustering methods. The XGBOOST algorithm identified MRPL17 as the most significant prognostic gene among those associated with stem cells. Additionally, the research explores the relationship between MRPL17 expression and immune cell infiltration. Immunofluorescence staining of LIHC tissue samples was conducted to evaluate the expression and prognostic value of MRPL17, as well as its correlation with KI67.

**Results:**

Through single-cell analysis, this study identified 14 essential stem cell-related genes, highlighting their significance in the diagnosis, prognostication, and potential treatment strategies for LIHC patients. Various machine learning algorithms indicated that MRPL17 is particularly associated with patient prognosis and responses to immunotherapy. Furthermore, experimental results demonstrate that MRPL17 is upregulated in LIHC and correlates with poor prognosis, as well as positively correlating with KI67.

**Conclusion:**

Cancer stem cells are pivotal in the mechanisms of immune evasion within the tumor microenvironment and have a substantial impact on treatment results. This study experimentally validated MRPL17 as a promising prognostic biomarker, emphasizing the need to target liver cancer stem cells to improve patient prognosis and enhance treatment effectiveness.

## 1 Introduction

Liver hepatocellular carcinoma (LIHC) is recognized as one of the most lethal malignant tumors worldwide (1). The World Health Organization has reported that liver cancer is the third leading cause of cancer-related deaths, particularly in developing nations and regions endemic to hepatitis, where both morbidity and mortality rates remain alarmingly high (2, 3). Despite recent progress in early detection, surgical interventions, and local ablation techniques, the insidious nature of LIHC, coupled with its nonspecific early symptoms, often results in diagnoses at advanced stages. This delay frequently leads to suboptimal treatment outcomes and a poor prognosis (4). Thus, there is an urgent need to discover new prognostic markers that can facilitate timely intervention and management during the early phases of the disease.

Currently, the most commonly employed prognostic assessment tools for liver cancer include alpha-fetoprotein (AFP), liver function tests, and imaging modalities (5). However, these traditional markers exhibit notable limitations in clinical practice. For example, not all patients with liver cancer present elevated AFP levels, and false positives may arise in certain benign liver conditions (6). Additionally, the heterogeneous nature of liver cancer makes it challenging for a single marker to adequately reflect a patient’s prognosis. Therefore, the establishment of diverse prognostic markers with clearer biological relevance is crucial for improving early detection rates of liver cancer and laying the groundwork for personalized therapeutic strategies. Recent advancements in single-cell sequencing technologies allow researchers to analyze the tumor microenvironment and its heterogeneity at a granular level, identifying gene expression patterns linked to the progression of liver cancer (7–9). This innovative approach provides new avenues for the identification of prognostic markers. By utilizing advanced data analysis techniques, such as machine learning, it is possible to extract potential biomarkers from complex genomic datasets, thereby predicting patient outcomes and treatment responses (10–12). This methodology not only assists clinicians in evaluating patient prognoses more accurately but also identifies new targets for innovative therapeutic development, thereby propelling the field of precision medicine in liver cancer.

A unique population of cells known as cancer stem cells is defined by their capability to self-renew and differentiate into various cell types (13). These cells are crucial in the processes of tumor initiation, progression, metastasis, and the emergence of drug resistance. In the context of liver cancer, cancer stem cells are critical contributors to recurrence and metastasis, making their functional and molecular study a key focus in contemporary tumor biology (14). These stem cells exhibit a range of characteristics, including high proliferation rates, resistance to drugs, and the capability to differentiate. Such traits enable liver cancer stem cells to survive in various microenvironments and endure external stresses, like chemotherapy and radiotherapy, ultimately leading to tumor recurrence (15). Furthermore, research shows that liver cancer stem cells engage in diverse interactions with different cell types found in the tumor microenvironment, which includes immune and stromal cells, through intricate signaling pathways that together promote tumor progression (16). As a result, therapeutic approaches focused on liver cancer stem cells hold potential for improving patient outcomes and lowering recurrence rates. Recently, immunotherapy has gained considerable attention in treating cancer, particularly methods that target the tumor microenvironment (17, 18). Despite the progress in immunotherapy research for liver cancer, the disease’s high heterogeneity and the intricate nature of the tumor microenvironment often lead to less than optimal treatment results. Studies indicate that cancer stem cells within the tumor microenvironment not only affect tumor biology but also influence the immune escape strategies utilized by these tumors (19). By secreting immunosuppressive substances and modifying the immune cell landscape in the tumor microenvironment, they obstruct the immune system’s ability to effectively attack the tumors. The distinct immune evasion tactics of these stem cells present significant obstacles for immunotherapy in liver cancer. Liver cancer stem cells can actively suppress T cell function by expressing various immunosuppressive factors (20). Furthermore, they can exacerbate the immunosuppressive environment by promoting the infiltration of regulatory T cells and tumor-associated macrophages. This immune evasion renders conventional immunotherapy less effective, leading to increased interest in immunotherapeutic approaches that specifically target liver cancer stem cells. The objective of our study is to pinpoint signature genes linked to stem cell markers via single-cell analysis and explore their implications for prognosis and immunotherapy in LIHC.

## 2 Materials and methods

### 2.1 Datasets and patient samples

This study analyzed two LIHC samples (GSM3064818 and GSM3064821) sourced from the GSE112271 dataset at the single-cell level. Additionally, we incorporated RNA sequencing data and clinical details from the TCGA-LIHC dataset. For the development and validation of diagnostic models, we utilized multiple datasets, including TCGA-LIHC, GSE45267, GSE39791, GSE112790, and GSE102079. Furthermore, we included 240 primary liver cancer samples from the IGCG database in our analysis. We employed LIHC tissue chips to investigate the expression and correlation of key genes. After excluding samples with incomplete clinical data and those lost to follow-up, a total of 92 LIHC tissue samples and 93 normal liver tissue samples were included in this study.

### 2.2 Processing of single-cell RNA-seq data

For single-cell analysis, we utilized four LIHC samples from the GSE112271 dataset (21). The Seurat package was employed to generate objects and filter out low-quality cells, ensuring that only high-quality data were included in our analysis. We conducted standard data preprocessing, examining the percentages of gene count, cell count, and mitochondrial content. Filtering criteria applied included the exclusion of genes detected in fewer than three cells and cells with fewer than 200 genes. Each cell’s UMI count was normalized using a scale factor of 10,000, standardizing the data across samples. After log transformation of the data, we applied the Seurat (v3.0.2) ScaleData function to further enhance the quality of the normalized data. The top 10 variable genes were selected for principal component analysis (PCA), identifying key genes contributing to dataset variability. We retained the first 11 principal components for UMAP visualization and clustering, which provided insights into the underlying structure of the data. Cell clustering was performed using the FindClusters function within the Seurat package, with a resolution set at 0.5 to ensure distinct clustering patterns among the cells.

### 2.3 Negative matrix factorization cluster analysis and differential expression analysis in the TCGA-LIHC dataset

The NMF algorithm was employed to identify biologically significant coefficients in the gene expression matrix, organizing genes and samples to emphasize the internal structural characteristics of the data, thereby facilitating sample grouping (22). Differential expression analysis comparing clusters A and B was conducted using the ‘Limma’ R package, applying criteria of |logFC| > 0.5 and an adjusted p-value of <0.05. Subsequently, the ‘NMF’ R package was utilized to cluster all samples based on differentially expressed genes (DEGs) identified within the subclusters, aiming to uncover potential molecular subtypes. The ‘brunet’ algorithm was applied with 100 iterations for each specified value and a range of 2 to 10 clusters. The optimal number of clusters was determined based on cophenetic correlation, dispersion, and silhouette width (23). The Limma package in R (version 3.40.2) was employed to analyze the differential expression of mRNA between cancerous and adjacent non-cancerous tissues in the TCGA-LIHC dataset.

### 2.4 Immune infiltration analysis

The immune microenvironment plays a crucial role in tumor progression and influences the effectiveness of cancer treatments (24, 25). To ensure the credibility of the immune score results, we utilized the immunedeconv R package (26). Extensive testing was performed on each algorithm, revealing unique advantages. The XCELL method was selected for this study due to its capacity to assess a broader range of immune cell types (27).

### 2.5 Constructing diagnostic model

We developed various diagnostic models related to LIHC by combining multiple machine learning algorithms. The training was conducted using the TCGA-LIHC dataset, with validation across the GSE45267, GSE39791, GSE112790, and GSE102079 datasets. Each combination was evaluated based on its area under the curve (AUC) value, with the best model selected based on the combination yielding the highest average AUC. Receiver operating characteristic (ROC) curve analysis was performed using the pROC package [1.18.0], and the results were visualized using ggplot2 [3.3.6].

### 2.6 Gene enrichment analysis

Genes associated with relevant pathways were collected and analyzed using the GSVA package in R. Single-sample gene set enrichment analysis (ssGSEA) was performed with the method parameter set to ‘ssgsea’. We examined the correlation between gene expression and pathway scores using Spearman correlation analysis.

### 2.7 Expression and prognostic relevance of MRPL17 in LIHC tissue microarrays analyzed by immunofluorescence methods

To prepare tissue sections, paraffin slices were immersed in two tanks of xylene for 15 minutes each, followed by sequential immersion in absolute ethanol, 95% ethanol, 85% ethanol, 75% ethanol, and distilled water, allowing 5 minutes for each solution. The sections were then placed in a repair box containing pH 9.0 EDTA alkaline antigen repair solution and heated in a pressure cooker for 2 minutes. After natural cooling, the sections were washed three times with PBS (pH 7.4) for 5 minutes each while shaking. They were then incubated in a 3% hydrogen peroxide solution at room temperature in the dark for 15 minutes. A blocking solution was applied dropwise to ensure even coverage of the tissue, and the sections were blocked at room temperature for 30 minutes. Subsequently, the MRPL17 antibody (bs-17773R), diluted with antibody diluent, was added to the sections and incubated overnight at 4°C. The following day, sections were washed three times with PBS for 5 minutes each, and after gently shaking the slices dry, a poly-HRP secondary antibody corresponding to the primary antibody species was applied dropwise and incubated at room temperature in the dark for 10-20 minutes (28, 29). The solution containing the TSA fluorescent dye must be uniformly distributed over the sections and allowed to incubate at room temperature for a duration of 15 minutes. Subsequently, apply the ready-to-use DAPI dye onto the sections, and incubate them in the dark at room temperature for 10 minutes. In the final step, mount the slides and obtain images using a fluorescence microscope. The intensity of immunostaining was rated on a scale from 0 to 3 to evaluate the strength of the reaction, while a second scale from 1 to 4 was utilized to measure the percentage of positive staining. To arrive at the final expression score, the intensity score was multiplied by the percentage scale score.

### 2.8 Statistical analysis

The expression levels of MRPL17 in both LIHC and normal tissues were assessed using the Wilcoxon rank-sum test. Prognostic analysis was conducted using the log-rank test. Spearman correlation analysis was applied to assess the correlation between gene expression and stemness scores. A p-value of less than 0.05 was established as the threshold for statistical significance.

## 3 Result

### 3.1 Screening for stem cell-related genes

**Figure 1:**
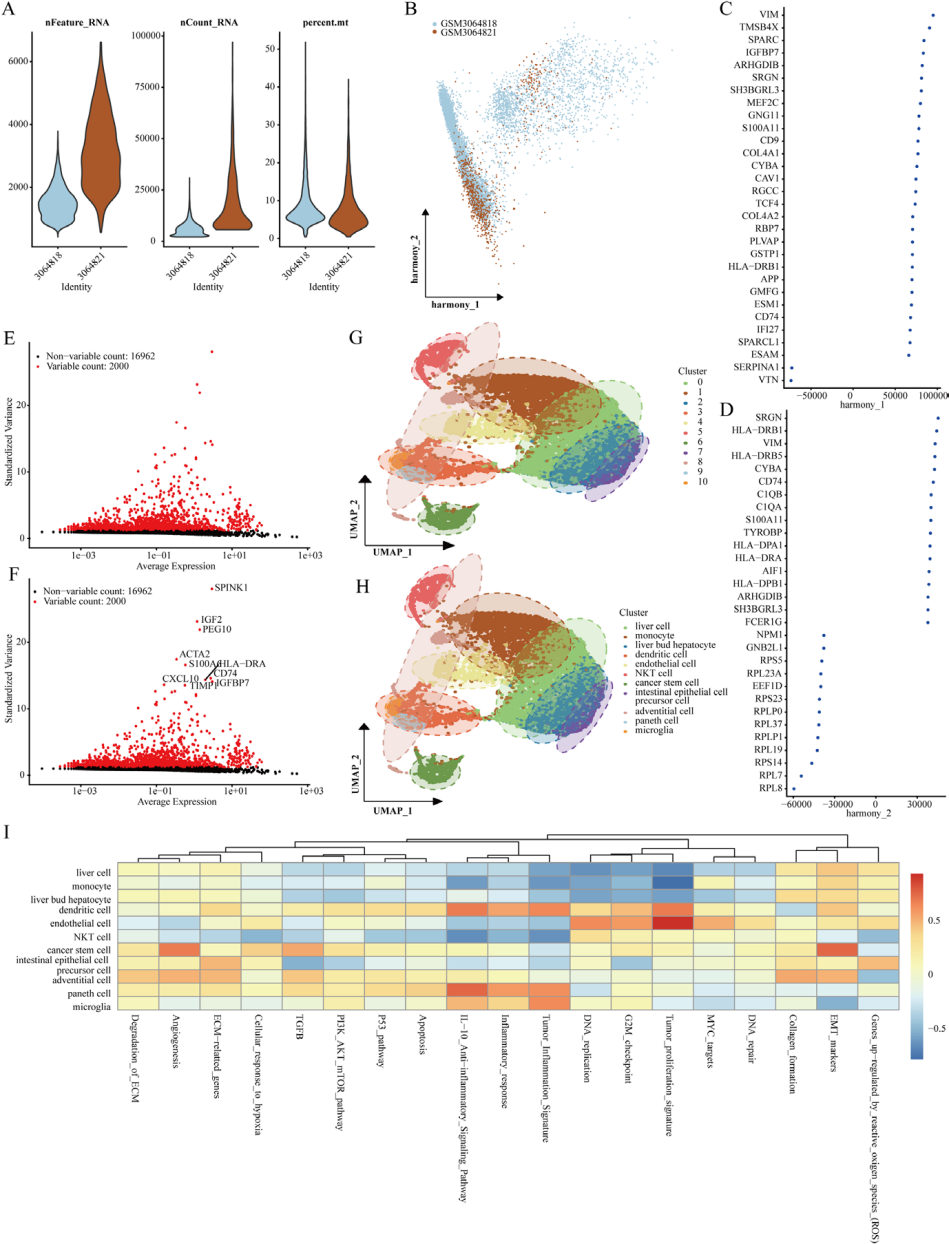
Identification of genes marking stem cells. **(A)** Quality assessment of scRNA-seq for various cell sub-populations. **(B–D)** PCA analysis visuals after the integrated elimination of batch effects. **(E, F)** Post-count batch removal conducted to identify highly variable genes. **(G, H)** Stratification of LIHC samples utilizing the UMAP technique. **(I)** Functional analysis of distinct cell populations.

Our analysis commenced with two LIHC samples sourced from the GSE112271 dataset, applying rigorous cell quality control standards: each cell had to contain at least 200 RNA molecules, no more than 6000 RNA molecules, and a maximum of 10% mitochondrial RNA (**Figure 1A**). Subsequently, we utilized the HARMONY technique to identify the highly variable genes from the filtered dataset, and we performed bulk deletion analysis based on these feature sets (**Figures 1B–D**). An ANOVA test highlighted the top 10 genes exhibiting significant differential expression within the cell samples: SPINK1, IGF2, PEG10, ACTA2, S100A6, CXCL10, HLA-DRA, CD74, TIMP1, and IGFBP7 (**Figure 1E, F**). The single-cell analysis categorized the two LIHC samples into 11 distinct cell groups, including microglia, Paneth cells, adventitial cells, intestinal epithelial cells, precursor cells, cancer stem cells, NKT cells, endothelial cells, dendritic cells, liver bud hepatocytes, monocytes, and liver cells (**Figures 1G, H**). Notably, functional analysis indicated that stem cell populations were associated with processes such as angiogenesis and epithelial-mesenchymal transition (EMT) (**Figure 1I**).

### 3.2 Screening for stem cell-related differential prognostic genes

**Figure 2:**
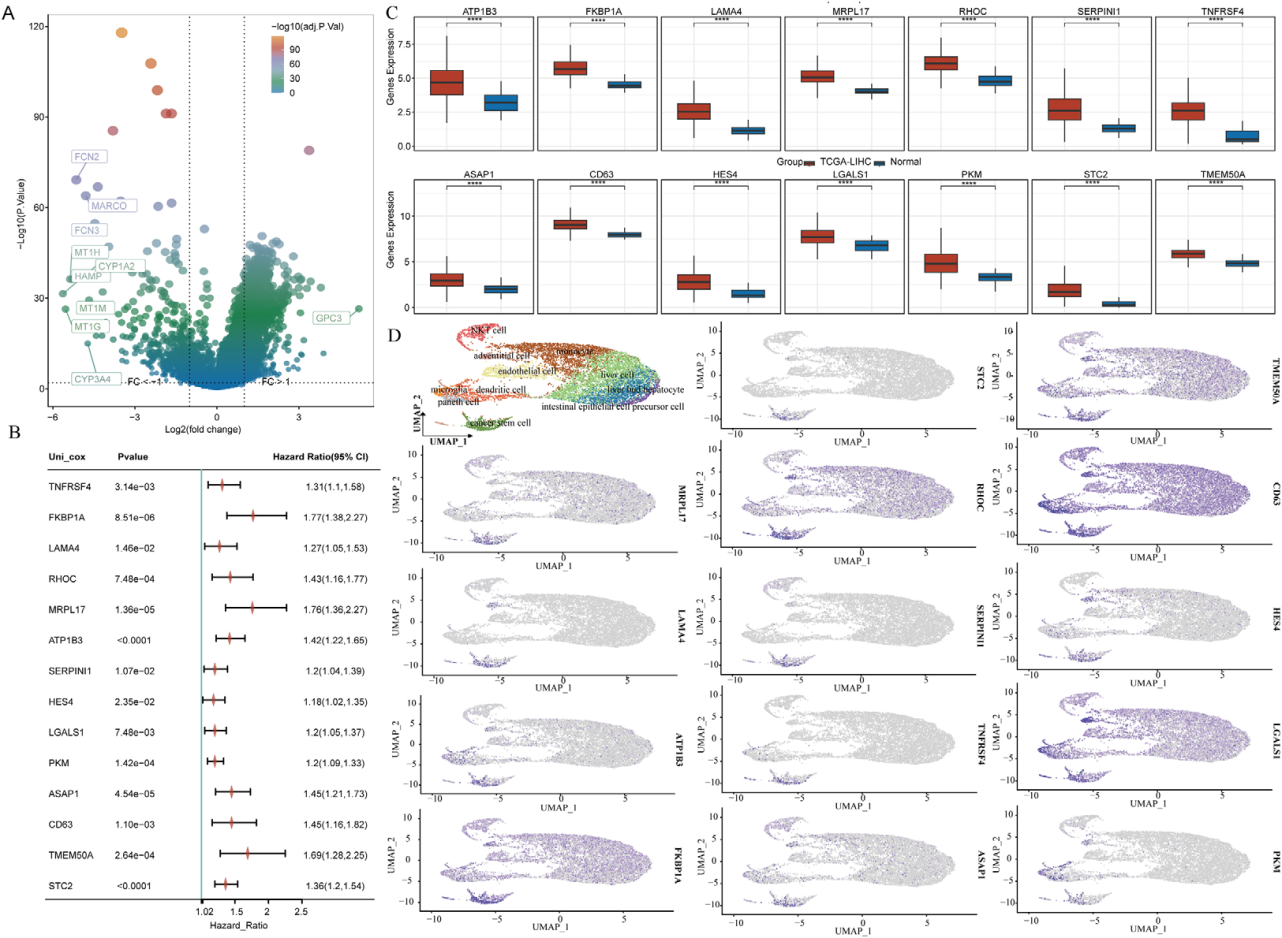
14 stem cell-related differential genes identified as associated with LIHC prognosis. **(A)** Volcano plot showing variance analysis. **(B)** Expression levels of differential genes associated with stem cells. **(C)** Prognostic evaluation of differential genes connected to stem cells. **(D)** Distribution of stem cell-related differential genes across various cell populations.

The analysis of differential expression, resulted in the identification of 2,452 genes that showed significantly increased expression in LIHC when compared to normal liver tissues (**Figure 2A**). Following this, we cross-referenced these genes with those previously recognized as stem cell-related, assessing their prognostic relevance. In the end, we pinpointed 14 distinct genes that presented stem cell characteristics linked to the prognosis of LIHC (**Figures 2B, C**). Additional investigations indicated the relative abundance of these 14 genes across various cell populations (**Figure 2D**).

### 3.3 NMF-based non-negative matrix clustering analysis

**Figure 3:**
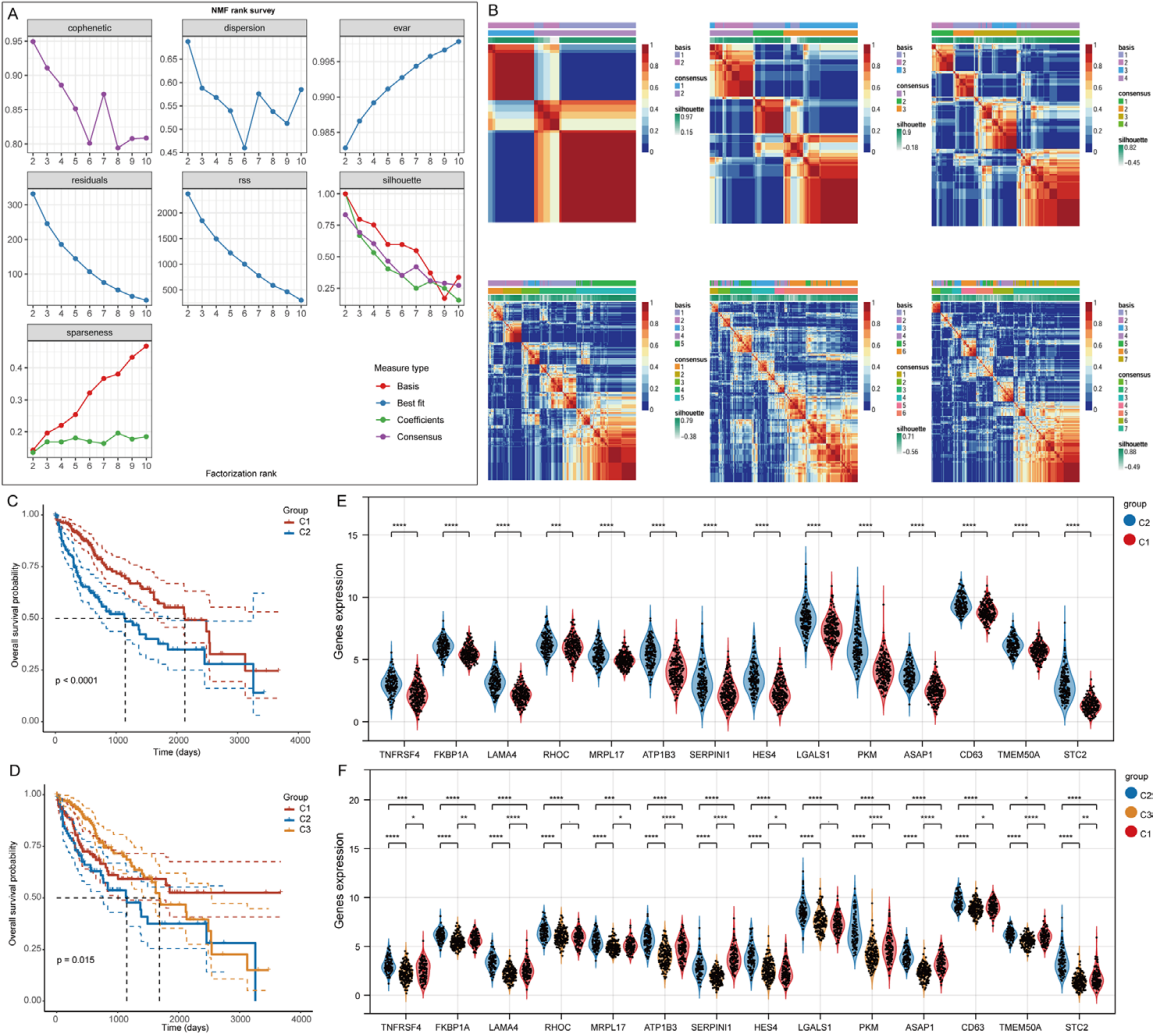
Clustering of LIHC samples based on NMF cluster analysis methods. **(A)** Evaluation of the performance and stability relating to the clusters via various methods. **(B)** Consensus map for NMF clustering results. **(C, D)** Variations in survival among the different clusters. **(E, F)** Gene expression differences associated with stem cells across the various clusters. *p< 0.05, **p< 0.01, ***p< 0.001, ****p< 0.0001.

For clustering the TCGA-LIHC samples, we employed the NMF clustering technique. We analyzed co-expression curves to identify the most appropriate method for partitioning the TCGA-LIHC sample subgroups. The best grouping was signaled by the point on the curve that exhibited the most significant decline in the co-expression metrics. Our analysis suggested that segmenting TCGA-LIHC samples into seven groups was most appropriate (**Figures 3A, B**). However, this division did not meet the analytical requirements for subsequent studies. Therefore, we opted to categorize the samples into two or three groups. Analysis of specific stem cell-related gene expression across these groups showed that when divided into two groups, cluster 1 exhibited a significantly better prognosis than cluster 2. When divided into three groups, cluster 1 had the best prognosis, while cluster 3 had the worst (**Figures 3C, D**). Notably, significant differences in gene expression were observed between the groups, with a more pronounced distinction in the two-cluster grouping (**Figures 3E, F**).

### 3.4 Functional analysis of stem cell-related genes

**Figure 4:**
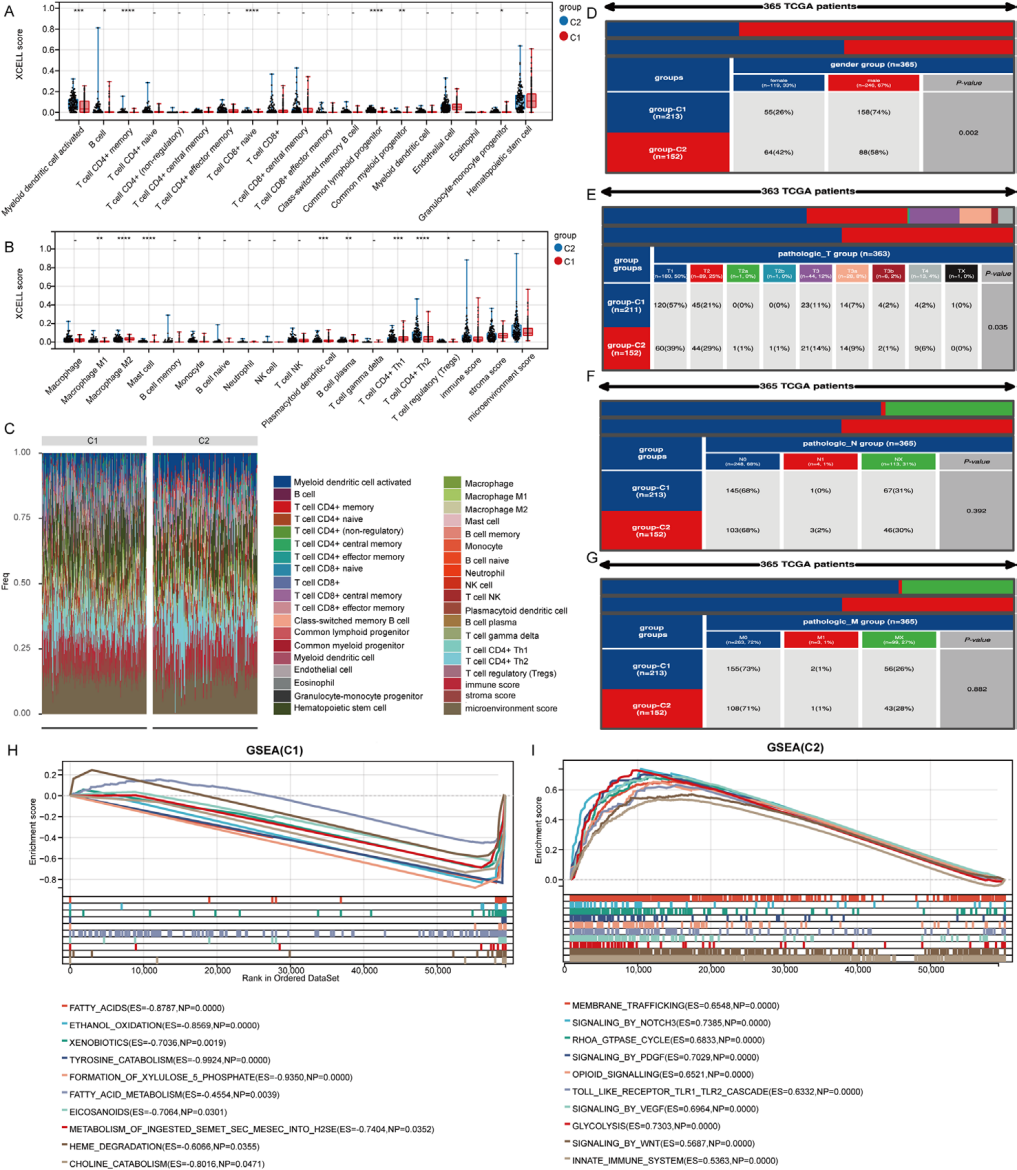
Genes marking stem cells are linked to immune cell infiltration. **(A, B)** Examination of genes associated with stem cells in relation to immune cell infiltration. **(C)** Heatmap displaying varying levels of infiltration by different immune cells. **(D-G)** Variations in the distribution of various subgroups across different pathological stages of LIHC. **(H, I)** Analysis of gene enrichment for two distinct clusters. *p< 0.05, **p< 0.01, ***p< 0.001, ****p< 0.0001.

To evaluate immune cell infiltration in the TCGA-LIHC samples, we analyzed signature genes associated with 35 immune cell types using the XCELL algorithm. Sixteen immune cell types exhibited significant differences in infiltration levels, indicating a close relationship between stem cell-related genes and immune cell infiltration in LIHC (**Figures 4A, B**). A heatmap illustrated the levels of immune cell infiltration across the two clusters (**Figure 4C**). Additionally, we examined patient distribution across various T stages, N stages, M stages, and genders, revealing differences in the distribution of patient numbers across T stages and gender groups in clusters 1 and 2 (**Figures 4D–G**). Analysis of gene enrichment in the two clusters indicated that cluster C1 was mainly connected to fatty acids, ethanol metabolism, and eicosanoid-related processes, whereas cluster C2 was associated with signaling pathways including WNT, PDGF, NOTCH3, and opioids (**Figures 4H, I**).

### 3.5 Construction of a diagnostic model

**Figure 5:**
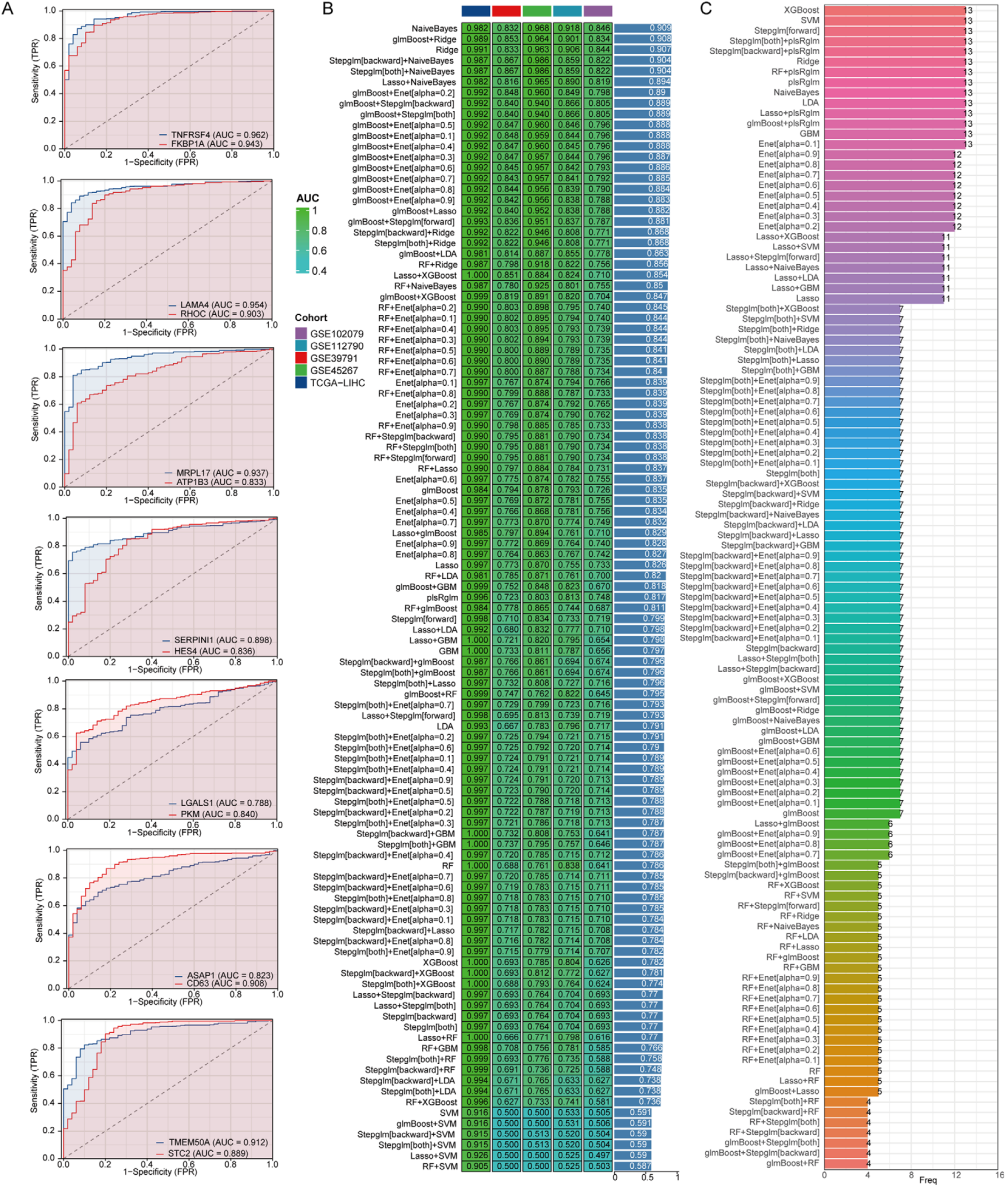
The combination of the NaiveBayes algorithm is regarded as the most effective arrangement for developing diagnostic models. **(A)** An analysis of the predictive capabilities of stem cell-related genes for diagnosing LIHC patients. **(B)** AUC values for diagnostic models generated from different combinations of algorithms. **(C)** The number of genes incorporated into diagnostic models that were created using various algorithm combinations.

To investigate the function of genes related to stem cells in LIHC, we evaluated their ability to predict LIHC diagnosis in patients by utilizing ROC curves. All genes analyzed demonstrated notable predictive potential for diagnosing LIHC, with AUC values surpassing 0.8 (**Figure 5A**). Consequently, we focused on developing diagnostic models using five datasets: the TCGA-LIHC dataset for training and GSE45267, GSE39791, GSE112790, and GSE102079 for validation. Among the algorithm combinations tested, the NaiveBayes algorithm proved most effective for model development, achieving an AUC value of 0.982 in the training set (TCGA-LIHC) and AUC values of 0.832, 0.968, 0.918, and 0.846 in the validation cohorts (GSE39791, GSE45267, GSE112790, and GSE102079). The average AUC value for the diagnostic model across all five datasets reached 0.909, indicating excellent predictive capability (**Figure 5B**). The diagnostic model constructed with the NaiveBayes algorithm included 13 genes associated with biochemical recurrence: TNFRSF4, LAMA4, RHOC, MRPL17, ATP1B3, SERPINI1, HES4, LGALS1, PKM, ASAP1, CD63, TMEM50A, and STC2 (**Figure 5C**).

### 3.6 Multiple machine learning algorithms identify the central role of MRPL17

**Figure 6:**
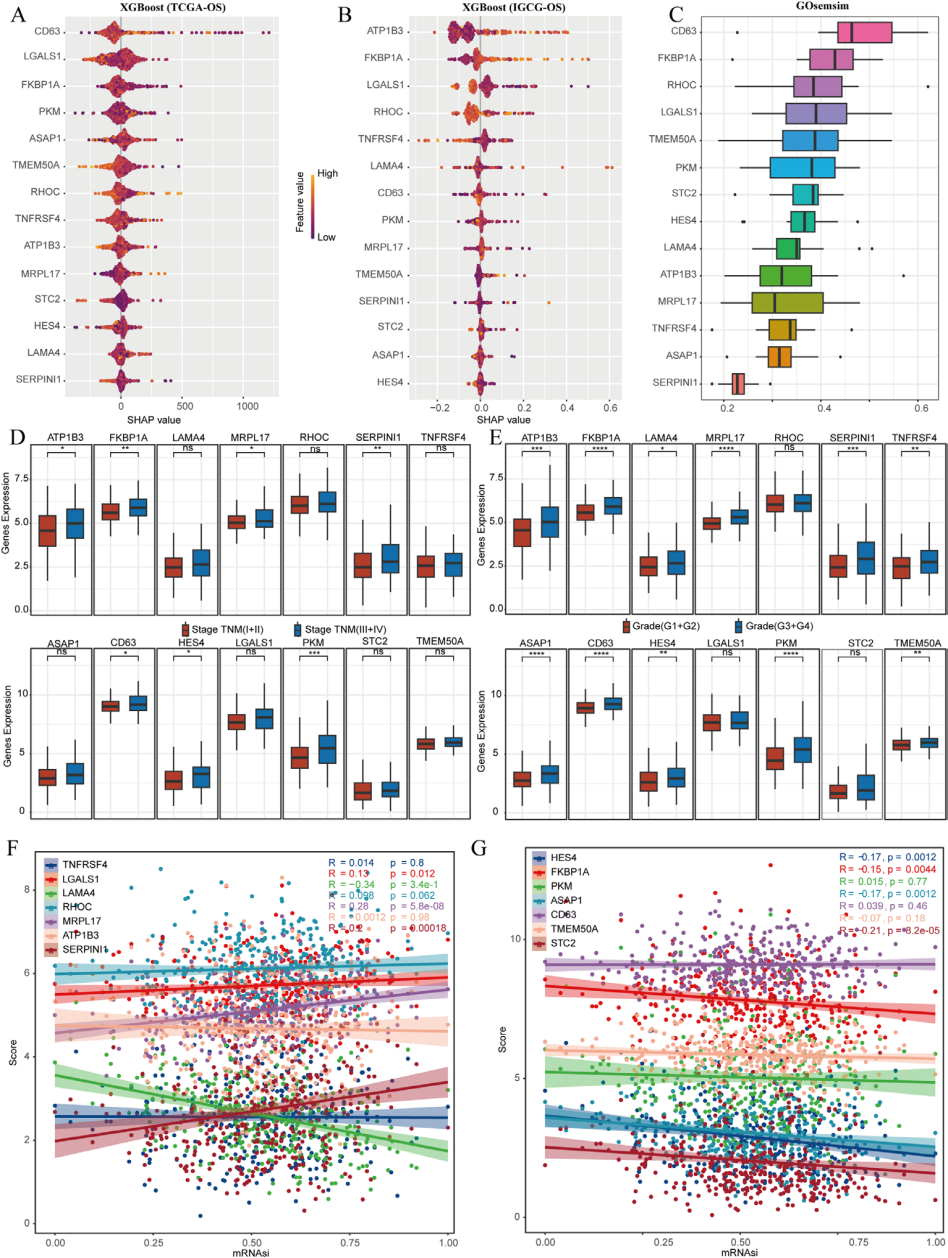
MRPL17 is the most critical gene for LIHC prognosis. **(A)** The XGboost algorithm identifies the 14 genes that are most significantly linked to OS in the TCGA-LIHC dataset. **(B)** Similarly, the XGboost algorithm determines the top 14 genes most related to OS in the IGCG-LIHC cohort. **(C)** Gosemsim analysis discovers key genes associated with stem cell-related functions. **(D, E)** The differential expression of genes linked to stem cells across various staging grades is demonstrated in the TCGA-LIHC dataset. **(F, G)** An analysis of the correlation between the stemness score and gene expression is provided. ns=p> 0.05, *p< 0.05, **p< 0.01, ***p< 0.001, ****p< 0.0001.

We analyzed stem cell-related genes in TCGA-LIHC and IGCG samples using the XGBoost algorithm to determine their association with OS. The top ten genes significantly associated with OS were CD63, LGALS1, FKBP1A, PKM, TMEM50A, RHOC, TNFRSF4, ATP1B3, and MRPL17 (**Figures 6A, B**). Gosemsim analysis was employed to rank these stem cell-related genes based on gene ontology similarities (**Figure 6C**). We examined expression differences of these 14 genes across various staging grades in the TCGA-LIHC dataset, finding significant differences for ATP1B3, FKBP1A, MRPL17, SERPINI1, CD63, HES4, and PKM (**Figures 6D, E**). To measure the relationships between these genes and tumor stemness, we employed the one-class logistic regression (OCLR) algorithm to determine mRNAsi, which serves as a marker for cell stemness obtained from gene expression data. Our investigation highlighted molecular signatures associated with cancer progression and prognosis through the differential expression of particular genes. Initially, we showcased stemness scores alongside the expression profiles of stem cell marker genes, and subsequently conducted a correlation analysis that indicated MRPL17 had the highest correlation with the stemness scores (**Figures 6F, G**). Taking these results into account, MRPL17 emerges as the most significant and important gene among stem cell marker genes for further studies on the progress of LIHC.

### 3.7 Analysis of the correlation of immune infiltration of MRPL17 in LIHC

**Figure 7:**
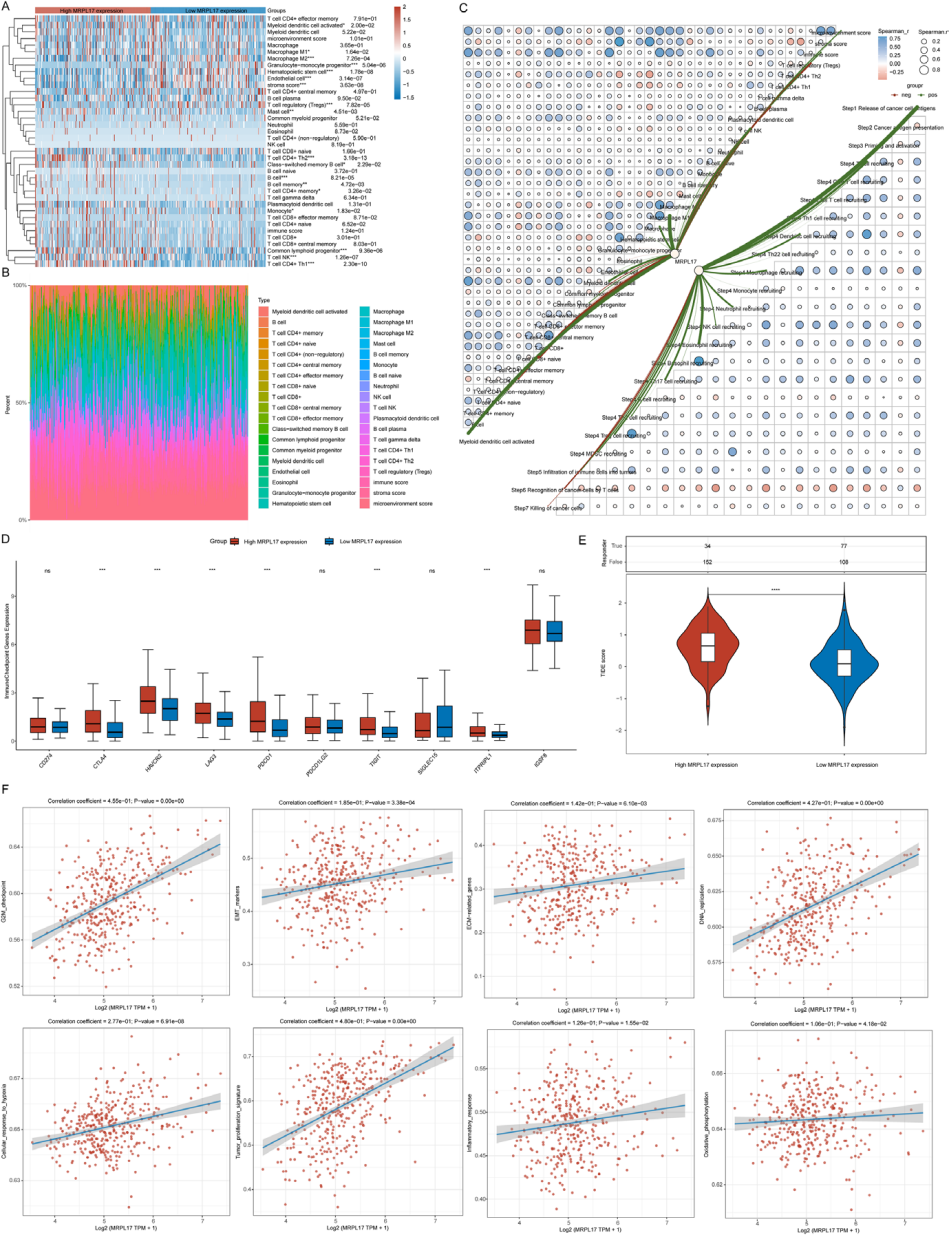
Analysis of MRPL17 Functionality. **(A)** Heatmap illustrating immune cell scores. **(B)** Proportion of immune cells infiltrating tumors across samples. **(C)** Diagram depicting the relationship between MRPL17 expression levels and the scores of immune cell infiltration. **(D)** Correlation of immune checkpoints within groups of high and low MRPL17 expression. **(E)** Variation in TIDE scores between MRPL17 high and low expression groups. **(F)** Functionality assessment of MRPL17 utilizing the TCGA-LIHC dataset. ns=p> 0.05, *p< 0.05, **p< 0.01, ***p< 0.001, ****p< 0.0001.

We sorted the samples from the TCGA-LIHC dataset according to the levels of MRPL17 expression to analyze variations in immune cell infiltration. Notable distinctions were found in the quantities of activated myeloid dendritic cells, M1 and M2 macrophages, granulocyte-monocyte progenitors, hematopoietic stem cells, endothelial cells, regulatory T cells (Tregs), mast cells, CD4+ Th2 T cells, and B cells (**Figure 7A**). We also illustrated the distribution of tumor-infiltrating immune cells across each TCGA-LIHC specimen (**Figure 7B**). A correlation network diagram depicted the association between MRPL17 expression and the fractions of immune cell infiltration, calculated via the XCELL and TIP algorithms, which included correlation analyses among various immune cell types (**Figure 7C**). We analyzed the expression of immune checkpoint-related genes in groups of high and low MRPL17 expression, discovering six genes with significant expression differences (**Figure 7D**). TIDE scores were computed for the high and low MRPL17 expression groups using the TIDE algorithm, revealing that patients with high MRPL17 expression exhibited elevated TIDE scores, suggesting less effective responses to immunotherapy (**Figure 7E**). Finally, through the ssGSEA algorithm, we assessed enrichment scores for multiple pathways to explore the association of MRPL17 expression with these pathways, revealing a positive relationship between MRPL17 expression and tumor proliferation as well as EMT (**Figure 7F**).

### 3.8 MRPL17 is highly expressed in LIHC

**Figure 8:**
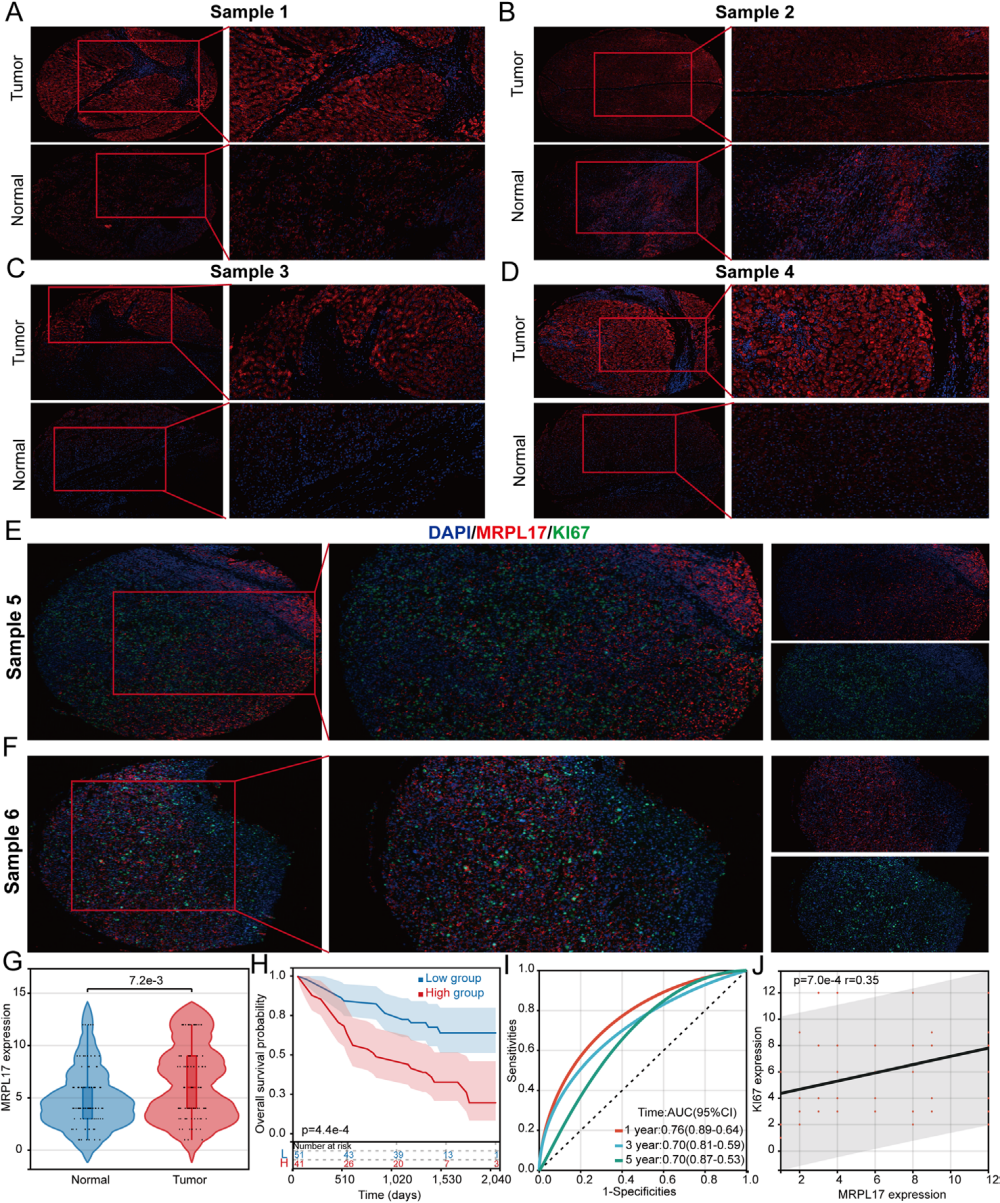
MRPL17 is highly expressed in LIHC. **(A-D)** Differential expression of MRPL17 in LIHC. **(E, F)** Correlation analysis between MRPL17 and KI67. **(G)** Violin plot of MRPL17 expression. **(H)** KM curve of overall survival of MRPL17. **(I)** Predictive ROC curve of MRPL17 expression on prognosis of LIHC patients. **(J)** Scatter plot of correlation between MRPL17 and KI67.

This study emphasizes the critical role of MRPL17 as a gene associated with LIHC stem cells. We collected 60 LIHC samples along with corresponding normal liver tissue samples for immunofluorescence staining to investigate differences in MRPL17 expression and its correlation with LIHC patient prognosis. Blue staining indicated cell nuclei, while red staining represented MRPL17 expression. Results indicated significantly elevated MRPL17 expression in LIHC compared to normal liver tissue (**Figures 8A–F**). Boxplots illustrated variations in MRPL17 expression between LIHC and normal tissues (**Figure 8G**). Furthermore, analysis revealed a correlation between MRPL17 expression and the prognosis of LIHC patients, suggesting that individuals with heightened MRPL17 levels experienced poorer outcomes (**Figures 8H, I**). MRPL17 is positively correlated with KI67 (**Figure 8J**).

## 4 Discussion

LIHC is a notably diverse malignant tumor, displaying significant variations in biological traits and clinical presentations across different patients. This study highlights the essential importance of liver cancer stem cells in the progression of the tumor, its recurrence, and resistance to therapies. These stem cells not only have the capability to self-renew and differentiate into several lineages, but they can also endure within the intricate tumor microenvironment and adjust to different types of stressors (30). The unique traits of liver cancer stem cells make them an essential focus for therapeutic strategies against liver cancer. Currently, the widespread use of single-cell technology has revealed numerous novel therapeutic targets among cell subtypes within tumor tissues (31, 32). In our research, we discovered signature genes linked to liver cancer stem cell markers utilizing single-cell RNA sequencing and non-negative matrix factorization cluster analysis. We also examined the potential implications of these findings for the prognosis and treatment of LIHC. This study provides fresh perspectives and possible biomarkers that could enhance early diagnosis, tailored therapies, and immunotherapeutic approaches for liver cancer.

Through an analysis at the single-cell level, our research uncovered 14 marker genes associated with stem cells, several of which have been documented before regarding their regulatory interactions with stem cells. For example, regulatory T cells expressing Tnfrsf4 facilitate the immune evasion of stem cells in chronic myeloid leukemia (33). Moreover, research has demonstrated that the suppression of RhoC expression can impede the proliferation, drug resistance, invasion, and metastasis of stem cells in ovarian cancer (34). In addition, it has been established that Hes4 plays a role in governing the proliferative characteristics of neural stem cells during the developmental stages of the retina (35). Assessments of the functionality of these cellular groups revealed a link between stem cell populations and angiogenesis. There exists a significant relationship between CSCs and the process of angiogenesis. On one hand, CSCs can play a direct role in tumor angiogenesis by releasing pro-angiogenic factors and differentiating into endothelial cells that construct blood vessels. On the other hand, the tumor vasculature not only supplies oxygen and nutrients but also produces factors that support CSCs, thus encouraging the generation of CSCs. In summary, a mutual enhancement occurs between tumor angiogenesis and stemness in tumors, which together contribute to treatment resistance and promote metastasis (36). Utilizing the expression patterns of the 14 identified stem cell-related genes, we implemented the NMF algorithm to conduct cluster analysis on LIHC samples sourced from the TCGA-LIHC dataset. Regardless of whether the LIHC samples were organized into two or three groups, there were notable differences in patient prognosis across the various groups. In order to explore the fundamental factors contributing to the variations in patient outcomes, we performed a gene enrichment analysis. The findings from this analysis indicated that several well-known regulatory pathways associated with tumor stemness were notably enriched in samples from cluster 2, including pathways such as VEGF, WNT, and PDGF signaling. This observation also clarifies why the samples within cluster 2 exhibit a poor prognosis.

In light of the lack of distinct diagnostic indicators for individuals suffering from LIHC, a significant number of patients are regrettably diagnosed at a later stage of the condition. To tackle this problem, our study aims to create diagnostic models for LIHC by employing various machine learning techniques. Within the training dataset, our model showcases impressive efficacy, achieving an AUC score of 0.982. In order to evaluate the effectiveness of our diagnostic method, we analyzed it through four varied datasets that consistently demonstrated the robustness and reliability of the model we developed. XGBOOST is an efficient boosted tree algorithm that is capable of handling large-scale datasets, demonstrating speed in both training and prediction. Numerous prior studies have indicated that XGBOOST typically offers superior prediction accuracy, particularly in the context of non-linear relationships. Utilizing the XGBOOST algorithm, we identified the ten most significant genes associated with OS in LIHC patients from a pool of 14 stem cell marker genes. By integrating the expression levels of these genes across various pathological stages and conducting correlation analyses with tumor stemness, we discovered that MRPL17 serves as an important stem cell marker gene pertinent to the prognosis and progression of LIHC. Functional analyses revealed that MRPL17 is connected to cell proliferation in LIHC, EMT, and oxidative phosphorylation. Numerous prior studies have recognized MRPL17 as a significant marker for tumor prognosis. For instance, Chengcheng et al. identified MRPL17 as a prognostic marker for lung cancer using bioinformatics analysis (37). Similarly, Miao et al. highlighted MRPL17 as a crucial pathogenic factor in endometrial cancer (38). However, its role in LIHC has not yet been reported.

Despite advances in liver cancer immunotherapy, treatment efficacy remains limited due to the significant heterogeneity and complex tumor microenvironment characteristic of this malignancy. Our study underscores the pivotal role of liver cancer stem cells in evading immune responses and offers new insights for the development of immunotherapeutic strategies. We conducted our research using multiple datasets from the TCGA-LIHC and GEO databases. Furthermore, while we confirmed the expression of MRPL17 in 92 LIHC tissue samples, the overall sample size is still inadequate. Larger-scale studies are necessary to validate the effectiveness of MRPL17 as a prognostic marker.

## 5 Conclusion

In conclusion, this research uncovered specific genes linked to liver cancer stem cells utilizing single-cell analysis alongside non-negative matrix decomposition clustering. Additionally, it examined their prospective roles in prognosis and immunotherapy. The results not only deepened our comprehension of liver cancer biology but also provided fresh perspectives for the creation of personalized therapies and novel immunotherapy approaches. Continued investigation is essential to further affirm the clinical relevance of these markers and to explore their potential uses in treating liver cancer.

## Data Availability

The original contributions presented in the study are included in the article/supplementary material. Further inquiries can be directed to the corresponding authors.
